# Association of Serum Vitamin D Level with Temporomandibular Disorder Incidence: A Retrospective, Multi-Center Cohort Study Using Six Hospital Databases

**DOI:** 10.3390/nu15132860

**Published:** 2023-06-24

**Authors:** Yeong-Gwan Im, Man-Yong Han, Hey-Sung Baek

**Affiliations:** 1 Department of Oral Medicine, Dental Science Research Institute, School of Dentistry, Chonnam National University, Gwangju 61186, Republic of Korea; imygwise@jnu.ac.kr; 2 Departments of Pediatrics, CHA Bundang Medical Center, CHA University School of Medicine, Seongnam 13496, Republic of Korea; 3 Department of Pediatrics, Kangdong Sacred Heart Hospital, Hallym University College of Medicine, Seoul 05355, Republic of Korea

**Keywords:** common data model, electronic health records, temporomandibular disorders, Vitamin D

## Abstract

The relationship between serum vitamin D levels and temporomandibular disorders (TMDs) remains unclear. Therefore, this study aimed to investigate the association between serum 25-hydroxyvitamin D (25[OH]D) levels and TMD incidence using large-scale health data. Clinical data from the electronic health records of six secondary or tertiary hospitals in Korea were used to evaluate the relationship between serum 25(OH)D levels and TMD incidence. The data were converted to the Observational Medical Outcomes Partnership Common Data Model. A cohort study was designed using the Cox proportional hazards model to examine the hazard ratio (HR) of TMD development after propensity score matching (PSM). An aggregate meta-analysis of the HR was subsequently performed. After 1:4 PSM, a target group with deficient 25(OH)D levels (<20 ng/mL) (N = 34,560) and comparator group with non-deficient 25(OH)D levels (≥20 ng/mL) (N = 47,359) were pooled from six hospital databases. HR meta-analysis demonstrated a significant association between deficient 25(OH)D levels and TMD incidence (pooled HR: 1.50; 95% confidence interval: 1.07–2.12). In conclusion, deficient 25(OH)D levels were found to be associated with an increased TMD risk. Therefore, vitamin D deficiency is a potential risk factor for TMD development.

## 1. Introduction

Vitamin D is a fat-soluble vitamin that plays a crucial role in diverse physiological processes in the body. One of its primary functions is to regulate the metabolism and absorption of calcium and phosphorus, which are essential minerals for bone health. In addition, vitamin D has been implicated in various other physiological processes, including immune function, cardiovascular health, and the prevention of certain types of cancer. Vitamin D deficiency is associated with an increased risk of several chronic diseases, including osteoporosis, type 2 diabetes, autoimmune diseases, and some types of cancer [1,2,3,4].

In the musculoskeletal system, vitamin D helps maintain the balance between bone formation and resorption, which is vital for the maintenance of bone density and strength [1,5]. Vitamin D also serves a key role in muscle-function regulation and has been shown to improve muscle strength and function, particularly in older adults [6,7]. This is considered to be a result of the fact that vitamin D receptors are present in skeletal muscle tissue as well as many other cells and tissues in the body [3], and vitamin D is involved in the regulation of muscle protein synthesis and calcium homeostasis [8]. Moreover, vitamin D deficiency has been implicated in the development of several musculoskeletal disorders, including osteoporosis, osteomalacia, and rickets. These conditions are characterized by reduced bone density, an increased risk of fractures, and muscle weakness and pain [1,5,9,10].

Temporomandibular disorders (TMDs) refer to a group of musculoskeletal conditions that affect the temporomandibular joint (TMJ), the masticatory muscles, and associated tissues [11]. According to the Diagnostic Criteria for Temporomandibular Disorders (DC/TMD) Axis I, TMDs can be categorized into Group I, which includes muscle disorders (such as myofascial pain with and without mouth opening limitation); Group II, which encompasses disc displacement with or without reduction and mouth opening limitation; and Group III, which comprises arthralgia, arthritis, and arthrosis [12]. TMDs potentially cause pain, discomfort, and difficulty in jaw function, such as chewing and opening the mouth. The overall TMD prevalence rates in the general population are 9.7%, 11.4%, and 2.6% for myalgia, disc displacements, and arthralgia, respectively [13]. Additionally, the prevalence of TMJ disorders is 11% in children and adolescents and 31% in adults and older individuals [14].

The etiology of TMDs is multifactorial, with various biological, psychological, environmental, and treatment-related factors playing a role. Some of the most commonly studied factors include genetic predisposition, hormonal factors, joint pathology, emotional stress, psychological conditions such as anxiety and depression, trauma, occlusal factors, parafunctional habits, and specific dental interventions on tissues [15,16,17,18]. TMD patients may present overlapping symptoms with other chronic pain conditions, including headache, fibromyalgia, and neurological disorders [19,20,21].

The management of TMDs encompasses patient education, biobehavioral therapy, pharmacologic therapy, physical therapy, oral appliance therapy, occlusal treatment, and surgical management [11]. Conservative treatment options, such as counseling, exercises, oral appliance therapy, and manual therapy, should be considered as the first-line therapy, and, in cases of severe acute pain or chronic pain, pharmacologic therapy, minimally invasive procedures, and surgical procedures may be necessary [22]. Recently, novel bioactive molecules and emerging therapeutic strategies, including intra-TMJ delivery systems, hold promise as innovative treatment approaches for TMDs [23].

Vitamin D has also been implicated in TMDs. Emerging evidence suggests a potential association between vitamin D deficiency and the development of TMDs. According to a recent systematic review [24], patients with TMDs commonly exhibit lower serum levels of vitamin D, and furthermore, the findings indicated a potential involvement of vitamin D receptor (VDR) polymorphisms in the development of TMDs. However, only a limited number of studies have investigated the association between vitamin D and TMDs, and the results have been inconsistent [18,25,26,27,28,29,30]. While certain studies have concluded that vitamin D is associated with TMDs [18,25,26], others have reported no such association [27,28,29]. Furthermore, few studies have examined this association using large sample sizes, and no long-term follow-up studies have investigated the relationship between the two.

Therefore, this study aimed to investigate the potential association between vitamin D deficiency and the incidence of TMDs. We hypothesized that lower serum vitamin D levels would be associated with an increased risk of developing TMDs. To achieve this objective, we conducted a retrospective multi-center cohort study using the Observational Medical Outcomes Partnership Common Data Model (OMOP-CDM). By leveraging the extensive and diverse data available within this model, we analyzed a large patient population and gathered comprehensive information on the relationship between serum vitamin D levels and TMDs.

## 2. Materials and Methods

### 2.1. Data Source

This study utilized hospital-based cohort data that were converted to the OMOP-CDM format using the FEEDER-NET platform, which provides electronic health record (EHR) data while protecting patients’ personal information. We collaborated with multiple secondary and tertiary hospitals in Korea. These institutions were part of distributed research networks in Korea that had implemented the OMOP-CDM. The participating institutions encompassed hospitals and healthcare systems with integrated EHRs or other structured healthcare databases. The multi-center, controlled cohort study included clinical data on 7,952,869 patients from six secondary or tertiary hospitals in Korea, and these data were converted to the OMOP-CDM version 5.3. The OMOP-CDM is a data structure that standardizes and integrates patient-level information, including healthcare visits, diagnoses, procedures, medications, and laboratory test results, while also ensuring patient anonymity. A diagnosis record in the OMOP-CDM includes a patient identifier, diagnosis date, diagnosis code, and the mapping and coding system, such as the International Classification of Diseases, Tenth Revision (ICD-10), linked to the OMOP-CDM Standardized Vocabularies [31]. During the mapping process, specific target vocabularies such as the Systematic Nomenclature of Medicine (SNOMED) for diagnoses, RxNorm for drugs, and Logical Observation Identifiers Names and Codes for other observations, such as laboratory results and vital sign measurements, are utilized [32].

This study included the following hospitals: (1) Pusan National University Hospital, Pusan (PNUH; 1,753,001 patients); (2) Gyeongsang National University Hospital, Changwon (GNUH; 618,246 patients); (3) Kyung Hee University Hospital, Seoul (KHMC; 2,010,456 patients); (4) Myongji Hospital, Goyang (MJH; 880,392 patients); (5) Wonkwang University Hospital, Iksan (WKUH; 1,001,794 patients); and (6) Daegu Catholic University Hospital, Daegu (DCMC; 1,688,980 patients). All hospitals signed a memorandum of understanding for research in border-free zones.

Enrollment periods were from 2011 to 2018 in PUNH, from 2009 to 2022 in GNUH, from 2008 to 2018 in KHMC, from 2003 to 2020 in MJH, from 1998 to 2018 in WKUH, and from 2005 to 2018 in DCMC. This study was approved by the Institutional Review Board (IRB) of Kangdong Sacred Heart Hospital (IRB number: 2019-09-005). The necessity for obtaining written informed consent was waived by the IRB. The affiliated hospitals, being part of the Research Border-Free Zone within the Korean CDM data network, acknowledge the IRB approval from the research-organizing center, thereby exempting the need for individual IRB approval.

### 2.2. Study Design and Cohort Definition

**Figure 1:**
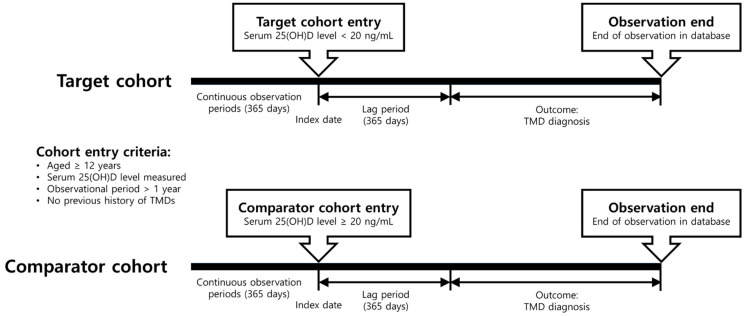
Study flow chart for the entire observation period.

This was a retrospective, observational, comparative, new-user cohort study that was conducted at multiple centers. The study design involved identifying participants who had undergone blood tests to measure 25-hydroxyvitamin D (25[OH]D) levels, with the index date being the date of blood-sample collection. The cohorts comprised individuals who had not been diagnosed with TMDs before at least one year from the index date. This study’s primary outcomes were the initial TMD diagnosis. The occurrence or diagnosis of TMDs was confirmed after the observation period of at least 365 days after the index date (i.e., lag period). Therefore, involved individuals without TMDs were monitored for TMD development for at least one year after entry into the study. Participants were either censored at the time of outcome identification or at the conclusion of the observation period in the database, whichever occurred first (Figure 1).

We excluded patients with a history of traumatic TMJ dislocation, TMJ fracture, infectious TMJ arthritis, or primary osteosarcoma of the jaw’s articular cartilage. The concept set expression included the following diagnoses: “temporomandibular joint-pain-dysfunction syndrome”, “temporomandibular joint internal derangement”, “temporomandibular joint disorders”, “temporomandibular joint disorder, unspecified”, “temporomandibular joint disorder”, “internal derangement of temporomandibular joint”, and “derangement of temporomandibular joint”. Excluded diagnoses were as follows: “traumatic dislocation of temporomandibular joint”, “traumatic closed dislocation of temporomandibular joint”, “traumatic arthritis of the temporomandibular joint”, “temporomandibular joint fracture”, “primary osteosarcoma of articular cartilage of jaw”, “open subluxation jaw”, “open division, temporomandibular ligament”, “open dislocation of jaw”, “injury of meniscus of temporomandibular joint”, “infectious arthritis of temporomandibular joint due to internal joint prosthesis”, “infectious arthritis of temporomandibular joint”, and “fracture dislocation of temporomandibular joint”.

**Figure 2:**
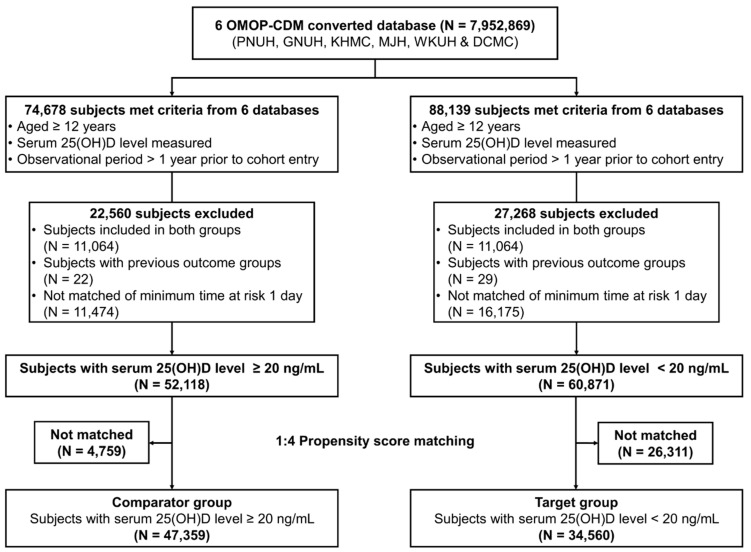
Study flow diagram displaying participant inclusion in the target and comparator cohort groups. OMOP–CDM: Observational Medical Outcomes Partnership–Common Data Model; N: number; PNUH: Pusan National University Hospital; GNUH: Gyeongsang National University Hospital; KHMC: Kyung Hee University Hospital; MJH: Myongji Hospital; WKUH: Wonkwang University Hospital; DCMC: Daegu Catholic University Hospital; 25(OH)D: 25-hydroxyvitamin D.

Participants were divided into two groups according to their serum 25(OH)D levels: deficient and non-deficient. The target group consisted of individuals with 25(OH)D deficiency, which was defined as a 25(OH)D level < 20 ng/mL, while the comparator group comprised individuals with non-deficiency (≥20 ng/mL), following the Endocrine Society Task Force on Vitamin D clinical practice guidelines [33]. This cutoff level has also been adopted as the recommended vitamin D requirement for virtually all normal, healthy individuals by various professional organizations and societies, with bone health as the primary basis for this recommendation [34]. Participants were excluded if they belonged to either group based on multiple 25(OH)D level tests. In total, 60,871 and 52,118 participants constituted the 25(OH)D-deficient and 25(OH)D-non-deficient groups, respectively (Figure 2).

### 2.3. Covariates

Demographic and clinical variables were considered covariates to balance the baseline characteristics between the 25(OH)D deficient and non-deficient groups. The target and comparator cohorts were compared based on several covariates, including age, gender, documented comorbidities (coded using standard medical terminologies such as SNOMED-CT or ICD-10), procedures (e.g., surgeries, treatments), prescribed medications (using drug terminologies such as RxNorm) within the 365 days preceding the index date, and the Charlson Comorbidity Index. Age at the index date and sex were included as demographic characteristics, as in Table S1. The specific covariates used in each hospital are shown in Table S2.

### 2.4. Statistical Analysis

This cohort study’s data were analyzed using the OHDSI CohortMethod R package, an open-source software for large-scale analytics, and Cyclops R package. ATLAS (version 2.7.5) was employed, and the analysis was performed using FEEDER-NET. Propensity score matching (PSM) was employed to control for potential confounding variables and enhance the validity of causal inference in this study. Large-scale PSM was performed using the OMOP-CDM tool. Logistic regression was used to estimate propensity scores, considering covariates such as age, gender, comorbidities, procedures, prescribed drugs, and the Charlson Comorbidity Index. The objective was to capture factors that could influence the outcomes of interest. PSM was performed using a 1:4 nearest-neighbor matching algorithm with a caliper width of 0.2 on the standardized logit scale. The balance of covariates between the unmatched and matched samples was assessed using standardized differences, considering differences > 10% as significant [35]. This approach aimed to identify the best matches based on the proximity of propensity scores. Additionally, standardized differences were employed to assess the covariate balance between the matched cohorts. These methods aimed to mitigate confounding by creating comparable groups and evaluating covariate balance. The assumptions of unconfoundedness and common support were considered during the analysis. After PSM, 34,560 and 47,359 participants comprised the target and comparator groups, respectively (Figure 2).

To estimate the effect of serum 25(OH)D levels on TMD development, a Cox proportional hazards model was fitted to the matched cohorts using the CohortMethod R package available on GitHub (https://github.com/OHDSI/CohortMethod, accessed on 2 August 2022). Hazard ratios (HRs) and 95% confidence intervals (CIs) were calculated for the outcomes of interest. The incidence rate was determined as the number of events per 1000 person-years (PY). Kaplan-Meier analysis was utilized to estimate the probability of survival for patients with TMDs throughout the follow-up period. The log-rank test was then performed to compare the survival curves between the target and comparator cohorts.

To assess the potential for bias due to unmeasured confounding, we established negative outcomes that were presumably disassociated with either the target or comparator cohorts. *p*-values were corrected by applying the empirical null distribution to the negative control outcomes’ point estimates. We assumed that the true relative risk of negative control outcomes between the target and comparator cohorts was 1.

A random-effects meta-analysis was also conducted without aggregating data from each hospital. We assessed study heterogeneity using the *I*^2^ statistic, where heterogeneity was considered significant when the *I*^2^ value exceeded 50% and *p*-value was less than 0.10. All analyses were performed using R statistical software (version 3.6.1; R Foundation for Statistical Computing) and the R meta-package (version: 6.5-0).

### 2.5. Sensitivity Analysis

To ensure the reliability of the findings, various Cox proportional hazard analyses were conducted. A stricter criterion was adopted, whereby the 25(OH)D-sufficient group was defined as those having a 25(OH)D level > 30 ng/mL, thus excluding individuals with insufficient serum 25(OH)D levels ranging from 20 to 30 ng/mL. In addition, a less stringent criterion based on a 30 ng/mL cutoff was used to form two groups: 25(OH)D-sufficient (≥30 ng/mL) and 25(OH)D-insufficient/deficient (<30 ng/mL).

## 3. Results

### 3.1. Participant Characteristics

In all hospital datasets after PSM, the target group, which was defined as the 25(OH)D-deficient group, included 34,560 participants, whereas the comparator group, which was defined as the 25(OH)D non-deficient group, included 47,359 participants. The matched cohorts’ baseline demographic and clinical data in each hospital are described in Tables S1 and S2, respectively. Prior to PSM, differences were noted between the target and comparator groups in terms of age-group distribution, sex ratio, medical history (e.g., acute respiratory disease and urinary tract infection), and medication history (e.g., use of antibiotics and anti-inflammatory drugs). However, after PSM, the age-group distribution, sex ratio, medical history, and medication history were balanced between the two groups. Each hospital exhibited slightly different characteristics. However, the 50s and 60s age groups accounted for the largest proportion, and the male-to-female ratio was comparable across all hospitals.

### 3.2. Primary Outcomes

**Figure 3:**
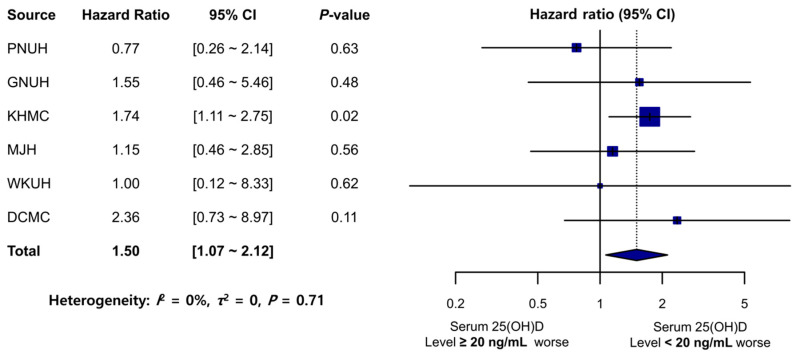
Forest plot of the risk of TMD development in the comparator (25[OH]D ≥ 20 ng/mL) and target (25[OH]D < 20 ng/mL) groups. PNUH: Pusan National University Hospital; GNUH: Gyeongsang National University Hospital; KHMC: Kyung Hee University Hospital; MJH: Myongji Hospital; WKUH: Wonkwang University Hospital; DCMC: Daegu Catholic University Hospital; 25(OH)D: 25-hydroxyvitamin D; CI: confidence interval.

The average follow-up duration for TMD incidence across hospitals was 2.5 years (Table 1). Remarkably, among the participants in the comparator group at KHMC (N = 21,029), 80 individuals developed new-onset TMDs, while in the target group (N = 14,997), 74 individuals had new-onset TMDs. This finding indicates a significantly higher likelihood of developing TMDs in the 25(OH)D-deficient group (HR: 1.74; 95% CI: 1.11–2.75; *p* = 0.02). The TMD incidence rate (per 1000 PY) was higher in the target group than in the comparator group for GNUH, KHMC, WKUH, and DCMC (Table 1). HRs were above 1.00 for GNUH, MJH, and DCMC, while for WKUH, the HR was 1.00. These results suggest an increased risk of TMDs in the target groups, although statistical significance was not achieved. However, the meta-analysis revealed a significant association between the 25(OH)D-deficient group and increased TMD risk (pooled HR: 1.50; 95% CI: 1.07–2.12) (Figure 3). No significant heterogeneity was observed across the databases (*I*^2^ = 0%, *τ*^2^ = 0, *p* = 0.71) (Figure 3).

### 3.3. Sensitivity Analysis

**Figure 4:**
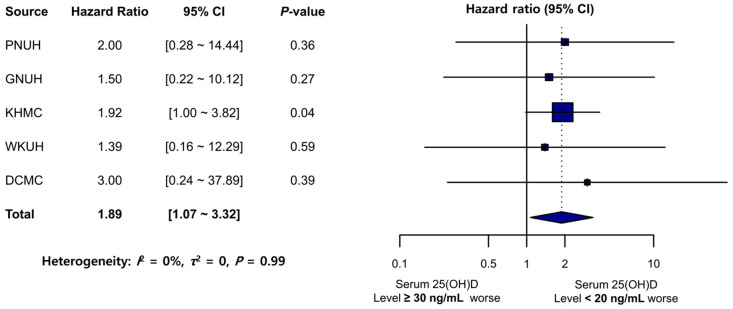
Forest plot of the risk of TMD development in the sufficient 25(OH)D (≥30 ng/mL) and deficient 25(OH)D (<20 ng/mL) groups. PNUH: Pusan National University Hospital; GNUH: Gyeongsang National University Hospital; KHMC: Kyung Hee University Hospital; WKUH: Wonkwang University Hospital; DCMC: Daegu Catholic University Hospital; 25(OH)D: 25-hydroxyvitamin D; CI: confidence interval.

The sensitivity analysis results, wherein a more stringent definition of a sufficient serum 25(OH)D level was applied, were consistent with those of the main analysis (Figure 4). The TMD incidence rate (per 1000 PY) in the 25(OH)D-deficient group (<20 ng/mL) exceeded that in the sufficient-25(OH)D group (≥30 ng/mL), except for that in MJH where valid data for between-group comparison were inaccessible. The meta-analysis revealed that the 25(OH)D-deficient group (<20 ng/mL) was significantly associated with an increased TMD risk (pooled HR: 1.89; 95% CI: 1.07–3.32). No significant heterogeneity was noted across the databases (*I*^2^ = 0%, *τ*^2^ = 0, *p* = 0.99).

**Figure 5:**
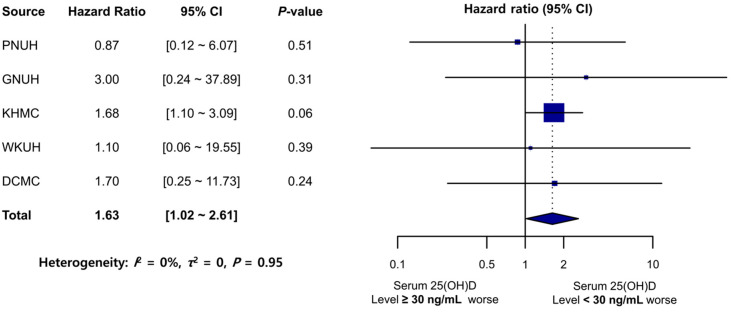
Forest plot of the risk of TMD development in the sufficient 25(OH)D (≥30 ng/mL) and insufficient/deficient 25(OH)D (<30 ng/mL) groups. PNUH: Pusan National University Hospital; GNUH: Gyeongsang National University Hospital; KHMC: Kyung Hee University Hospital; WKUH: Wonkwang University Hospital; DCMC: Daegu Catholic University Hospital; 25(OH)D: 25-hydroxyvitamin D; CI: confidence interval.

Another sensitivity analysis results, wherein a less stringent criterion with the 25(OH)D level cutoff of 30 ng/mL was applied, were consistent with those of the primary analysis (Figure 5). The TMD incidence rate (per 1000 PY) in the 25(OH)D-insufficient/deficient group (<30 ng/mL) exceeded that in the sufficient-25(OH)D group (≥30 ng/mL), except for that in MJH. The meta-analysis revealed that the 25(OH)D-insufficient group (<30 ng/mL) was significantly associated with an increased TMD risk (pooled HR: 1.63; 95% CI: 1.02–2.61). No significant heterogeneity was noted across the databases (*I*^2^ = 0%, *τ*^2^ = 0, *p* = 0.95).

## 4. Discussion

The present study provides valuable information supporting a potential association between vitamin D deficiency and an increased risk of developing TMDs based on multi-center EHR data in Korea. The study’s findings indicate that individuals with a serum vitamin D level < 20 ng/mL carry a 1.5 times higher risk of developing TMDs than those with a 25(OH)D level ≥ 20 ng/mL. This result suggests that low serum vitamin D levels may be a contributing or predisposing factor in TMD occurrence.

This study observed an association between deficient 25(OH)D levels and TMD incidence across different datasets and hospitals utilizing the OMOP-CDM. Although not statistically significant in some hospitals, most hospitals showed HRs above 1 for the 25(OH)D-deficient group, indicating an increased risk of developing TMDs. These variations may be attributed to differences in patient populations, including demographics, medical history, and medication usage, as well as variances in data collection and documentation practices among the hospitals. However, despite these variations, the meta-analysis of the combined data from all hospitals revealed a significant association between deficient 25(OH)D levels and increased risk of TMD development. This finding suggests that while there may be some heterogeneity among the individual hospitals, the overall trend supports the hypothesis that vitamin D deficiency is associated with a higher risk of TMDs.

Several reasons potentially explain the relationship between vitamin D and TMDs. Vitamin D plays a critical role in regulating the metabolism of calcium and parathyroid hormone, which are essential for maintaining bone homeostasis and cartilaginous metabolism in the TMJ. Low vitamin D levels are significantly associated with the progression of TMJ osteoarthritis in both young and post-menopausal women [26]. Additionally, vitamin D has been reported to possess anti-inflammatory functions by reducing proinflammatory-cytokine production and inhibiting T-cell response [36]. Furthermore, studies have indicated that genetic factors play a role in the development of TMDs, with particular emphasis on the VDR gene [37,38]. The VDR gene is crucial in mediating the biological effects of vitamin D through its intracellular receptor protein. Numerous VDR gene polymorphisms have been identified, which can affect the activity of the VDR protein and its association with various diseases, including TMDs [39,40,41]. Specifically, the *BsmI* variant of the VDR gene has been suggested to be linked to TMDs, such as disc displacement with reduction [25]. A recent systematic review has also presented evidence supporting the involvement of VDR polymorphisms in TMD patients [24]. These findings suggest that vitamin D levels and variations in the VDR gene may contribute to the development and progression of TMDs, although the underlying mechanisms remain incompletely understood.

The findings of the present study are consistent with several previous investigations, supporting a significant association between vitamin D deficiency and TMDs. Nemati et al. [18] observed a notable disparity in serum vitamin D levels between the TMD group (55 participants) and the healthy control group (55 participants). Yildiz et al. [25] demonstrated significant differences in serum vitamin D levels between the patient (104 participants) and control (102 participants) groups, with a higher prevalence of severe vitamin D deficiency among TMD patients. Hong et al. [26] reported significant variations in serum 25(OH)D levels among the control, initial TMJ osteoarthritis, and severe TMJ osteoarthritis groups in young women, with a significant association between TMJ osteoarthritis progression and vitamin D levels in both young and post-menopausal women. However, Demir et al. [27] and Madani et al. [28] found no significant differences between TMD patients and control subjects regarding vitamin D levels. Conversely, Staniszewski et al. [30] discovered lower vitamin D levels in the control group (60 participants) compared to the TMD group (60 participants).

Most prior clinical studies examining the link between vitamin D deficiency and TMDs in humans have been limited by small sample sizes of 100–200 individuals and cross-sectional designs, leading to weak and inconclusive associations [18,25,26,27,28,30]. In contrast, this study used a multi-center retrospective longitudinal design, which provides more robust evidence. As for the strengths of this study, a large sample of 7,952,869 Korean patients was used, and PSM was applied to control for confounding variables. The use of the OMOP-CDM database enabled patient diversity among participants, enhancing the assessment of the association between vitamin D deficiency and TMD development. However, the analysis of the CDM database did not yield information regarding the mean age and mean serum 25(OH)D levels, preventing direct comparisons of these parameters with other studies.

This study used the OMOP-CDM and employed PSM to address potential confounding factors and ensure a balance between the target and comparator groups. The matched cohorts’ baseline characteristics (Table 1) revealed that variables such as age-group distribution, sex ratio, medical history, and medication history were well-balanced after PSM. This demonstrates the effectiveness of PSM in achieving balance for these variables. However, PSM may not completely eliminate all group imbalances, especially for variables not included in the matching process or those with limited availability in the dataset. Despite our efforts to include relevant baseline characteristics in the propensity score model, there might still have been residual confounding factors that could impact the interpretation of the results. To address this concern, we performed sensitivity analyses by considering different cutoff values for serum 25(OH)D levels. This process ensured the robustness of the findings and enabled the evaluation of the potential impact of residual imbalances on the results. The consistent associations observed across different cutoff values suggest that the PSM achieved a reasonable balance, thereby minimizing the possible influence of residual imbalances on our interpretations.

This study included patients with available data within the OMOP-CDM. This may introduce selection bias and limit the generalizability of our findings to populations or settings with different data availability or healthcare practices. To enhance the generalizability of our findings, future studies should be conducted in diverse populations and settings, considering factors such as geographic location, ethnicity, socioeconomic status, and healthcare system characteristics. Replication studies with larger sample sizes and broader inclusion criteria would provide a more comprehensive understanding of the association between serum vitamin D levels and TMDs across different populations.

The present study utilized EHR data from hospital-based cohorts, and these data were converted to the OMOP-CDM format. Notably, the diagnoses recorded in the ICD-10 codes used in this study may not always match internationally accepted diagnostic classification criteria, such as DC/TMD [12]. For instance, the concept set expression in the OMOP-CDM format does not include terms such as myalgia and myofascial pain of the jaw muscles as well as arthralgia and degenerative joint disease of the TMJ, as defined by DC/TMD. Additionally, diagnoses recorded in the ICD-10 codes are potentially influenced by factors such as coding practices, reimbursement rules, and administrative requirements rather than solely reflecting the patient’s clinical presentation and diagnosis. Therefore, considering the potential limitations and biases introduced by ICD-10 code usage is imperative.

Serum vitamin D concentrations are known to vary seasonally, with lower levels observed during winter due to reduced synthesis in the body caused by decreased ultraviolet radiation. Therefore, failure to adjust for seasonal variations in serum vitamin D levels in this study might have limited the interpretation of the results. Previous studies have also demonstrated the impact of seasonal variations in serum vitamin D levels on study outcomes. For example, one study found that during winter months, lower serum vitamin D levels were associated with a higher risk of osteoporosis and related disorders [42]. Another study found that during seasons of lower serum vitamin D levels, the incidence of mental health disorders, such as depression, was higher [43]. Therefore, future studies investigating the relationship between vitamin D deficiency and TMDs should consider adjusting for seasonal variations in serum vitamin D levels.

This study has several limitations that need to be acknowledged. First, as an observational study, residual confounding factors might have influenced the results, despite using PSM. Second, the study did not adjust for seasonal variations in serum vitamin D levels, which could have affected the findings. Third, information regarding anthropometric indices was not available, and this might have influenced the results. Obesity can significantly impact various aspects, including vitamin D levels, as it affects both vitamin D synthesis and storage [33]. Factors related to obesity, such as reduced sunlight exposure, altered metabolism, and inflammation, can contribute to vitamin D deficiency [33]. However, our study did not address these specific factors, which is an important limitation to consider when interpreting our findings. Fourth, not all cohort participants were adequately diagnosed to meet the TMD diagnostic criteria. In 1992, the research diagnostic criteria for TMD (RDC/TMD) were established to allow clinicians to adopt a common diagnostic item for TMD patients, and in 2014 they were updated, resulting in the DC/TMD Axis I and II [12]. In this study, in some hospitals (PNUH, KHMC, and WKUH), patients were presumed to have been diagnosed with TMD by an orofacial pain specialist but not in other hospitals. Reliability could be questioned if the diagnosis of TMD was not based on specific diagnostic criteria or if a doctor performed it without expertise and experience in the field. Fifth, using ICD-10 codes for diagnoses might have introduced potential limitations and biases. Finally, this study is observational and cannot establish causality.

However, this study possesses several notable strengths. To our knowledge, it represents the first longitudinal multi-center investigation with a large sample size, utilizing big data to explore the association between serum 25(OH)D levels and the development of TMDs.

In conclusion, this multi-center EHR- and PSM-based study revealed an association between deficient 25(OH)D levels and an increased TMD risk and offers valuable information regarding the association between vitamin D deficiency and TMD development. This finding emphasizes the need for clinicians to consider an assessment of vitamin D levels in TMD diagnosis.

Future research should involve more hospitals and patients, measure serum vitamin D levels several times, observe for a longer period, and select individuals diagnosed according to the diagnostic criteria for TMDs as participants. It would be more desirable to classify TMD into subtypes, such as joint and muscle disorders, and to establish a relationship with vitamin D. Furthermore, research should explore underlying mechanisms and evaluate the effectiveness of vitamin D supplementation in TMD prevention and treatment.

## Figures and Tables

**Table 1:** Comparison of risk of TMD development between participants with serum 25(OH)D levels ≥ 20 ng/mL and those with levels < 20 ng/mL.

Hospital	Study Period	Number of Subjects (N = 81,919)		Patient-Year (Mean Follow-Up Time *)		Number of Outcome Events (TMD Diagnosis)		Incidence Rate per 1000 Patient-Years		HR	95% CI	*p*-Value
		Serum 25(OH)D Level ≥ 20 ng/mL (N = 47,359)	Serum 25(OH)D Level < 20 ng/mL (N = 34,560)	Serum 25(OH)D Level ≥ 20 ng/mL	Serum 25(OH)D Level < 20 ng/mL	Serum 25(OH)D Level ≥ 20 ng/mL	Serum 25(OH)D Level < 20 ng/mL	Serum 25(OH)D Level ≥ 20 ng/mL	Serum 25(OH)D Level < 20 ng/mL			
PNUH	2011–2018	9354	7054	23,182 (2.478)	16,723 (2.371)	26	11	1.12	0.66	0.77	0.26–2.14	0.63
GNUH	2009–2022	3152	2019	10,620 (3.369)	6584 (3.261)	11	7	1.04	1.06	1.55	0.46–5.46	0.48
KHMC	2008–2018	21,029	14,997	51,754 (2.461)	37,380 (2.492)	80	74	1.55	1.98	1.74	1.11–2.75	0.02
MJH	2003–2020	5344	3950	14,273 (2.671)	9533 (2.413)	17	11	1.19	1.15	1.15	0.46–2.85	0.56
WKUH	1998–2018	3615	2555	7755 (2.145)	5864 (2.295)	5	5	0.64	0.85	1.00	0.12–8.33	0.62
DCMC	2005–2018	4865	3985	11,841 (2.434)	9209 (2.311)	8	11	0.73	1.19	2.36	0.73–8.97	0.11

TMDs: temporomandibular disorders; HR: hazard ratio; CI: confidence interval; PNUH: Pusan National University Hospital; GNUH: Gyeongsang National University Hospital; KHMC: Kyung Hee University Hospital; MJH: Myongji Hospital; WKUH: Wonkwang University Hospital; DCMC: Daegu Catholic University Hospital. * Mean follow-up time = patient-year/number of subjects (unit: year).

## Data Availability

The datasets used and analyzed during the current study are available from the corresponding author on reasonable request.

## Supplementary Materials

The following supporting information can be downloaded at: https://www.mdpi.com/article/10.3390/nu15132860/s1, Table S1. Participant baseline characteristics in the multi-center OMOP-CDM database; Table S2. Participant clinical characteristics in the study cohorts.
