# Supplementary material for: Association of Serum Vitamin D Level with Temporomandibular Disorder Incidence: A Retrospective, Multi-Center Cohort Study Using Six Hospital Databases
Source: Nutrients. 2023 Jun 24;15(13):2860. doi: 10.3390/nu15132860 (PMC10343618; doi:10.3390/nu15132860)
Supplement: Supplementary file 1 [file nutrients-15-02860-s001.zip › nutrients-2386915-supplementary.pdf]

Table S1. Participant baseline characteristics in the multicenter OMOP-CDM database.

%	Matched cohort after PS adjustment								
	PNUH			GNUH			KHMC		
	Serum 25(OH)D level ≥ 20 ng/mL (N = 9,354)	Serum 25(OH)D level < 20 ng/mL (N = 7,054)	SD %	Serum 25(OH)D level ≥ 20 ng/mL (N = 3,152)	Serum 25(OH)D level < 20 ng/mL (N = 2,019)	SD %	Serum 25(OH)D level ≥ 20 ng/mL (N = 21,029)	Serum 25(OH)D level < 20 ng/mL (N = 14,997)	SD %
**Age group**									
12–14 years	0.5	0.4	-0.01	0.3	0.7	0.06	0.8	1.0	0.02
15–19 years	0.4	0.6	0.02	1.6	1.5	0.00	1.2	1.3	0.01
20–24 years	0.4	0.5	0.01	2.7	2.3	-0.02	1.9	1.7	-0.01
25–29 years	1.2	1.2	0.01	2.3	1.9	-0.03	2.0	1.8	-0.01
30–34 years	4.1	4.4	0.02	2.8	2.8	0.00	3.1	3.2	0.01
35–39 years	6.2	6.1	0.00	3.6	3.7	0.00	3.8	3.8	0.00
40–44 years	8.0	7.7	-0.01	4.5	4.0	-0.02	4.4	4.5	0.01
45–49 years	11.0	10.8	0.00	5.5	5.7	0.01	7.1	6.8	-0.01
50–54 years	14.2	14.6	0.01	9.2	10.2	0.03	10.4	10.7	0.01
55–59 years	16.3	16.3	0.00	13.3	13.2	0.00	13.2	14.1	0.02
60–64 years	14.3	14.8	0.01	15.4	13.2	-0.06	14.5	13.8	-0.02
65–69 years	9.8	9.8	0.00	11.5	12.3	0.03	11.9	12.2	0.01
70–74 years	7.1	6.7	-0.02	11.3	11.5	0.00	11.1	11.2	0.00
75–79 years	4.3	4.0	-0.02	8.6	9.2	0.02	8.6	8.0	-0.02
80-84 years	1.7	1.4	-0.02	5.4	5.2	-0.01	4.8	4.4	-0.02
85-89 years	0.4	0.6	0.02	1.6	2.1	0.04	1.2	1.1	-0.01
90-94 years	0.1	< 0.1	-0.02	0.4	0.4	0.01	0.3	0.3	-0.01
Sex									
Female	57.6	57.7	0.00	57.0	56.5	-0.01	57.5	58.1	0.01

%	MJH			WKUH			DCMC		
	Serum 25(OH)D level ≥ 20 ng/mL (N = 5,344)	Serum 25(OH)D level < 20 ng/mL (N = 3,950)	SD %	Serum 25(OH)D level ≥ 20 ng/mL (N = 3,615)	Serum 25(OH)D level < 20 ng/mL (N = 2,555)	SD %	Serum 25(OH)D level ≥ 20 ng/mL (N = 4,865)	Serum 25(OH)D level < 20 ng/mL (N = 3,985)	SD %
Age group									

12–14 years	1.2	1.3	0.01	-0.1	0.2	0.02	0.9	0.8	0.00
15–19 years	1.4	1.2	-0.01	0.6	1.3	0.08	0.4	0.7	0.04
20–24 years				0.6	1.4	0.08	0.2	0.3	0.02
25–29 years	2.2	2.0	-0.01	0.8	1.8	0.09	1.2	1.3	0.00
30–34 years	4.0	4.1	0.00	1.4	1.8	0.03	2.8	2.3	-0.03
35–39 years	9.7	10.3	0.02	2.1	2.9	0.05	5.1	5.0	0.00
40–44 years	7.5	7.1	-0.02	4.1	4.6	0.02	7.4	7.5	0.00
45–49 years	8.1	8.7	0.02	6.6	7.6	0.04	11.7	11.8	0.00
50–54 years	11.4	12.2	0.02	8.3	9.2	0.03	16.1	15.9	-0.01
55–59 years	13.2	11.6	-0.05	11.6	11.3	-0.01	15.6	15.8	0.01
60–64 years	10.0	10.5	0.02	14.2	11.5	-0.08	13.5	13.9	0.01
65–69 years	7.9	8.1	0.00	11.6	11.8	0.01			
70–74 years	6.9	7.0	0.01	10.9	10.5	-0.01	7.1	7.3	0.01
75–79 years	7.6	7.5	0.00	12.2	10.5	-0.05	4.8	4.6	-0.01
80-84 years	4.9	4.6	-0.01	10.0	8.6	-0.05	2.7	2.2	-0.04
85-89 years	2.3	2.0	-0.02	3.9	3.9	0.00	0.7	0.8	0.01
90-94 years	0.5	0.7	0.03	0.9	1.0	0.00	-0.1	-0.1	-0.01
Sex									
Female	54.6	55.5	0.02	55.2	56.0	0.02	53.6	52.9	-0.01

OMOP-CDM: Observational Medical Outcomes Partnership-Common Data Model; N: number; PS: propensity score; SD: standard difference; PNUH: Pusan National University Hospital; GNUH: Gyeongsang National University Hospital; KHMC: Kyung Hee University Hospital; MJH: Myongji Hospital; WKUH: Wonkwang University Hospital; DCMC: Daegu Catholic University Hospital.

Table S2. Participant clinical characteristics in the study cohorts.

%	Matched cohort after PS adjustment								
	PNUH			GNUH			KHMC		
	Serum 25(OH)D level ≥ 20 ng/mL (N = 9,354)	Serum 25(OH)D level < 20 ng/mL (N = 7,054)	SD %	Serum 25(OH)D level ≥ 20 ng/mL (N = 3,152)	Serum 25(OH)D level < 20 ng/mL (N = 2,019)	SD %	Serum 25(OH)D level ≥ 20 ng/mL (N = 21,029)	Serum 25(OH)D level < 20 ng/mL (N = 14,997)	SD %
**Medical history**									
Acute respiratory disease	0.4	0.6	0.02	3.8	4.4	0.03	1.7	1.6	-0.01
Chronic liver disease	0.6	1.0	0.04	2.4	2.9	0.03	2.0	2.0	0.00
Chronic obstructive lung disease	1.7	1.9	0.02	3.8	4.1	0.01	1.3	1.4	0.01
Crohn's disease	0.1	< 0.2	0.00	0.5	0.7	0.04	0.7	0.9	0.02
Dementia	0.3	0.2	-0.02	1.3	1.1	-0.02	1.7	2.0	0.02
Depressive disorder	1.9	1.6	-0.02	8.7	8.5	-0.01	3.2	3.2	0.00
Diabetes mellitus	5.3	5.0	-0.01	3.9	4.1	0.01	8.3	8.5	0.01
Gastroesophageal reflux disease	3.3	3.7	0.02	1.8	2.1	0.02	9.1	9.3	0.01
Gastrointestinal hemorrhage	0.4	0.5	0.02	1.2	1.6	0.04	1.6	1.5	0.00
Hyperlipidemia	4.7	4.6	-0.01	-0.1	-0.2	0.01	22.9	23.8	0.02
Hypertensive disorder	3.7	3.9	0.01	1.6	1.6	0.00	21.1	21.5	0.01
Lesion of liver	8.3	7.9	-0.02	6.2	6.0	0.00	1.6	1.6	0.00
Obesity	0.7	1.1	0.04	2.1	2.3	0.02	0.4	0.4	0.01
Osteoarthritis	0.2	0.5	0.05	1.7	1.4	-0.02	7.6	7.5	0.00
Pneumonia	0.9	0.5	-0.05	3.4	3.5	0.01	1.3	1.1	-0.02
Psoriasis	1.1	0.9	-0.02	0.4	0.2	-0.02	1.2	0.9	-0.02
Renal impairment	0.2	0.2	0.01	7.2	6.5	-0.03	2.8	2.8	0.00
Rheumatoid arthritis	0.7	0.9	0.03	3.9	3.4	-0.03	0.9	0.8	0.00
Schizophrenia	0.2	< 0.2	-0.02	2.4	2.5	0.00	0.2	0.2	0.00
Ulcerative colitis	< 0.1	< 0.2	0.03	1.0	0.9	-0.01	0.6	0.6	0.00
Urinary tract infectious disease	0.4	0.6	0.02	0.6	0.6	0.00	1.3	1.2	-0.01
Viral hepatitis C	0.5	0.6	0.01	0.8	0.8	0.01	0.2	0.1	0.00
Visual system disorder	3.6	3.4	0.00	9.8	9.0	-0.03	6.6	6.6	0.00
Medication use									
Agents acting on the renin-angiotensin system	5.6	5.9	0.02	22.4	21.7	-0.02	16.0	16.4	0.01
Antibacterials for systemic use	16.3	15.6	-0.02	31.8	32.1	0.01	22.5	22.3	0.00
Antidepressants	8.1	7.9	-0.01	25.9	25.3	-0.01	8.9	8.9	0.00
Antiepileptics	5.3	5.2	0.00	15.1	13.4	-0.05	11.1	11.1	0.00
Anti-inflammatory and antirheumatic products	13.4	13.3	0.00	27.2	26.7	-0.01	38.7	38.9	0.00
Antineoplastic agents	4.3	4.1	-0.01	7.4	7.4	0.00	14.8	15.0	0.01
Antipsoriatics	0.8	0.5	-0.03				3.6	3.3	-0.02
Antithrombotic agents	10.4	10.1	-0.01	21.9	21.5	-0.01	23.1	23.1	0.00
Beta blocking agents	5.3	5.3	0.00	17.6	16.3	-0.04	13.3	13.7	0.01
Calcium channel blockers	6.1	6.1	0.00	20.9	20.9	0.00	15.7	15.8	0.00
Diuretics	5.2	5.3	0.01	15.5	14.9	-0.02	12.1	11.9	-0.01

Drugs for acid-related disorders	32.9	33.8	0.02	51.9	53.1	0.02	46.8	46.7	0.00
Drugs for obstructive airway diseases	4.4	4.5	0.00	25.8	27.0	0.03	19.4	19.6	0.00
Drugs used in diabetes	4.3	4.5	0.01	21.9	21.0	-0.02	10.9	11.0	0.00
Immunosuppressants	1.3	1.5	0.02	5.0	4.8	-0.01	8.3	8.1	-0.01
Lipid modifying agents	8.4	7.8	-0.02	25.6	25.9	0.01	23.9	24.4	0.01
Opioids	35.3	36.1	0.02	31.9	32.0	0.00	25.2	25.0	0.00
Psycholeptics	41.5	43.6	0.04	41.8	38.9	-0.06	25.1	25.6	0.01
Psychostimulants, agents used for ADHD and nootropics	0.6	1.0	0.04	2.1	1.7	-0.03	2.0	58.1	0.01

%	MJH			WKUH			DCMC		
	Serum 25(OH)D level ≥ 20 ng/mL (N = 5,344)	Serum 25(OH)D level < 20 ng/mL (N = 3,950)	SD %	Serum 25(OH)D level ≥ 20 ng/mL (N = 11,165)	Serum 25(OH)D level < 20 ng/mL (N = 5,853)	SD %	Serum 25(OH)D level ≥ 20 ng/mL (N = 4,865)	Serum 25(OH)D level < 20 ng/mL (N = 3,985)	SD %
Medical history									
Acute respiratory disease	3.1	3.4	0.01	2.0	1.3	-0.05	1.9	1.9	-0.01
Chronic liver disease	1.2	1.1	-0.01	1.8	2.0	0.01	1.1	1.2	0.01
Chronic obstructive lung disease	0.6	0.6	0.00	0.9	1.2	0.03	0.5	0.7	0.02
Crohn's disease							0.5	0.7	0.02
Dementia	3.9	3.8	-0.01	5.8	5.6	-0.01	0.1	0.2	0.02
Depressive disorder	6.5	6.3	-0.01	4.8	4.7	-0.01	0.5	0.6	0.01
Diabetes mellitus	10.1	10.3	0.01	11.9	11.4	-0.01	1.3	1.1	-0.01
Gastroesophageal reflux disease	16.2	15.9	-0.01	3.0	2.9	-0.01	5.3	5.1	-0.01
Gastrointestinal hemorrhage	0.6	0.7	0.00	1.5	1.4	0.00	6.0	5.7	-0.01
Hyperlipidemia	25.1	25.5	0.01	21.2	21.4	0.01	0.7	0.6	-0.02
Hypertensive disorder	21.2	20.7	-0.01	36.9	35.4	-0.03	12.7	12.4	-0.01
Lesion of liver	1.0	1.1	0.00	3.0	2.5	-0.03	11.1	9.9	-0.04
Obesity	0.6	1.1	0.05				1.4	1.7	0.03
Osteoarthritis	1.8	2.0	0.01	2.0	2.7	0.05	0.2	0.6	0.05
Pneumonia	1.8	1.2	-0.04	3.3	3.1	-0.01	1.5	1.5	0.00
Psoriasis	0.4	< 0.1	-0.05	0.1	0.3	0.03	1.2	1.2	0.00
Renal impairment	2.7	2.6	0.00	22.6	22.3	-0.01	0.3	0.2	-0.02
Rheumatoid arthritis	0.8	0.9	0.01	0.2	0.3	0.01	4.2	3.1	-0.06
Schizophrenia	1.4	1.3	0.00	0.2	-0.2	-0.03	0.1	-0.1	-0.02
Ulcerative colitis				0.2	0.2	0.00	-0.1	-0.1	0.01
Urinary tract infectious disease	1.5	1.4	0.00	1.8	2.4	0.04	0.9	0.7	-0.02
Viral hepatitis C	0.2	0.2	0.00	0.3	0.2	-0.02	0.2	0.2	0.00
Visual system disorder	8.6	8.2	-0.01	7.8	7.7	0.00	6.7	6.2	-0.02
Medication use									
Agents acting on the renin-angiotensin system	16.9	17.2	0.01	32.5	31.4	-0.02	8.7	8.4	-0.01
Antibacterials for systemic use	25.6	23.5	-0.05	39.5	35.9	-0.07	13.1	11.8	-0.04

Antidepressants	18.6	16.9	-0.04	37.1	33.9	-0.07	5.9	5.8	0.00
Antiepileptics	9.5	9.4	0.00	15.2	14.2	-0.03	5.7	5.3	-0.02
Anti-inflammatory and antirheumatic products	32.5	31.8	-0.02	44.3	40.5	-0.08	22.0	22.4	0.01
Antineoplastic agents	5.7	5.6	0.00	14.3	15.0	0.02	11.4	11.3	0.00
Antipsoriatics	0.9	0.8	0.00	1.7	1.4	-0.02	0.8	0.7	-0.02
Antithrombotic agents	20.9	19.8	-0.03	39.7	36.7	-0.06	9.8	9.5	-0.01
Beta blocking agents	10.0	10.2	0.01	16.8	16.2	-0.02	6.2	6.1	0.00
Calcium channel blockers	15.1	14.5	-0.02	29.9	28.6	-0.03	7.6	6.9	-0.03
Diuretics	11.6	11.2	-0.01	22.2	19.7	-0.06	9.0	8.1	-0.03
Drugs for acid-related disorders	36.8	35.5	-0.03	57.4	54.8	-0.05	9.0	8.1	-0.03
Drugs for obstructive airway diseases	11.8	11.1	-0.02	25.5	23.8	-0.04	32.0	31.6	-0.01
Drugs used in diabetes	16.3	16.7	0.01	25.9	24.5	-0.03	11.3	10.6	-0.02
Immunosuppressants	3.1	3.0	-0.01	6.5	7.3	0.03	5.8	5.2	-0.03
Lipid modifying agents	24.4	24.2	0.00	36.5	35.8	-0.02	12.2	11.8	-0.01
Opioids	32.3	30.8	-0.03	38.4	35.4	-0.06	41.1	42.0	0.02
Psycholeptics	37.3	37.1	0.00	33.1	30.3	-0.06	61.5	62.2	0.02
Psychostimulants, agents used for ADHD and nootropics	2.0	1.9	-0.01	6.3	5.5	-0.03	1.1	0.7	-0.04

N: number; PS: propensity score; ADHD: Attention deficit hyperactivity disorder; PNUH: Pusan National University Hospital; GNUH: Gyeongsang National University Hospital; KHMC: Kyung Hee University Hospital; MJH: Myongji Hospital; WKUH: Wonkwang University Hospital; DCMC: Daegu Catholic University Hospital.
